# Gallic acid inhibition of Src-Stat3 signaling overcomes acquired resistance to EGF receptor tyrosine kinase inhibitors in advanced non-small cell lung cancer

**DOI:** 10.18632/oncotarget.10581

**Published:** 2016-07-13

**Authors:** Ai N.H. Phan, Tuyen N.M. Hua, Min-Kyu Kim, Vu T.A. Vo, Jong-Whan Choi, Hyun-Won Kim, Jin Kyung Rho, Ki Woo Kim, Yangsik Jeong

**Affiliations:** ^1^ Department of Biochemistry, Wonju College of Medicine, Yonsei University, Wonju, Gangwon-do, Republic of Korea; ^2^ Department of Global Medical Science, Institute of Lifestyle Medicine, Nuclear Receptor Research Consortium, Wonju College of Medicine, Yonsei University, Wonju, Gangwon-do, Republic of Korea; ^3^ Department of Convergence Medicine, Asan Medical Center, University of Ulsan, College of Medicine, Seoul, Republic of Korea; ^4^ Department of Pharmacology, Wonju College of Medicine, Yonsei University, Wonju, Gangwon-do, Republic of Korea

**Keywords:** gallic acid, Src, Stat3, EGFR-TKI resistance, lung cancer

## Abstract

Tyrosine kinase inhibitors (TKIs) targeting epidermal growth factor receptor (EGFR) have clinically benefited to lung cancer patients harboring a subset of activating EGFR mutations. However, even with the remarkable therapeutic response at the initial TKI treatment, most lung cancer patients eventually have relapsed aggressive tumors due to acquired resistance to the TKIs. Here, we report that 3, 4, 5-trihydroxybenzoic acid or gallic acid (GA), a natural polyphenolic compound, shows anti-tumorigenic effects in TKI-resistant non-small cell lung cancer (NSCLC). Using both *in vitro* growth assay and *in vivo* xenograft animal model, we demonstrated tumor suppressive effect of GA was more selective for the TKI-resistant cancer compared to the TKI-sensitive one. Mechanistically, GA treatment inhibited Src-Stat3-mediated signaling and decreased the expression of Stat3-regulated tumor promoting genes, subsequently inducing apoptosis and cell cycle arrest in the TKI-resistant lung cancer but not in the TKI-sensitive one. Consistent with the *in vitro* results, *in vivo* xenograft experiments showed the TKI-resistant tumor-selective growth inhibition and suppression of Src-Stat3-dependent signaling in the GA-treated tumors isolated from the xenograft model. This finding identified an importance of Src-Stat3 signaling cascade in GA-mediated tumor-suppression activity and, more importantly, provides a novel therapeutic insight of GA for advanced TKI-resistant lung cancer.

## INTRODUCTION

Lung cancer is a fatal disease leading to the highest number of cancer deaths worldwide [1, 2]. Despite advances in diagnosis and standard therapy, the treatment and prevention of lung cancer still requires a better understanding of the molecular mechanisms for cancer pathogenesis and development [1]. A targeted therapy blocking oncogenic EGFR is currently the only biological anti-cancer adjuvant strategy after surgery of lung cancer. As EGFR activation induces downstream signaling pathways driving tumor proliferation and/or anti-apoptosis, targeting activated EGFR signaling by using TKIs such as gefitinib or erlotinib markedly suppresses tumor growth, leading to remarkable clinical benefit for lung cancer patients. However, patients eventually acquire resistance to gefitinib or erlotinib, even with dramatic therapeutic response at the initial TKI treatment for EGFR-mutant NSCLC, leads to a clinical challenge for overcoming TKI resistance [3, 4]. Several clinical approaches to overcome TKI-resistant (TKIR) tumor progression have been developed, including radiation therapy for the region surgically resected, the combination of radiation and cytotoxic chemotherapy, or the development of next generation TKIs [4]. Second- and third-generation of TKIs irreversibly inhibit EGFR and successfully suppress T790M-haboring tumors [5–7]. In particular, the third-generation one (CO-1686 and AZD-9291) selectively inhibits activating EGFR mutants including T790M in an irreversible manner but sparing wild-type EGFR [8]. However, regardless of the advanced clinical settings, to overcome TKI-resistance still remains to be clinical and pharmacological challenges mainly due to adverse side effects as well as unclear biological resistant mechanisms [4].

Diverse biological factors contributing to the TKI-resistance include EGFR amplification or a secondary T790M mutation conferring the higher binding affinity of adenosine triphosphate to the receptor that eventually leads to stronger EGFR signaling. Alternatively, bypass pathways accounting for approximately 20% of TKI-resistance involve MET amplification resulting in activation of ERBB3/PI3K/AKT axis, elevated insulin-like growth factor 1 receptor (IGF1R) or ERK signaling by irreversible TKIs [9–11]. Activation of Stat3 signaling via feedback mechanism involving IL6R/JAK1/JAK2/Stat3 cascade has been recently reported as an additional TKIR mechanism. Activated Stat3 signaling, as a consequence, drives tumor growth and underlines resistance to TKIs in advanced lung cancer [12–14].

Some polyphenolic phytochemicals such as resveratrol, curcumin, EGCG, and GA are well-known natural compounds for anti-cancer effects [15–17]. Due to their low toxicity and antioxidant properties, many dietary supplements have been investigated as a preventive strategy for cancer [18]. However, functions of these natural compounds in the drug resistant NSCLC remain poorly understood. GA has been previously shown to suppress cancer growth by inducing apoptosis, inhibiting angiogenesis, or blocking lipopolysaccharide-induced nuclear factor-κB signaling in several types of cancers [19, 20].

Here, we found GA suppressed lung tumor growth, and of more interest, the growth inhibition of GA are more selective for the TKIR cancer cells compared to the TKI-sensitive (TKIS) cells. Mechanistically, we demonstrated that GA suppression of Src-mediated Stat3 signaling is important for the anti-tumorigenic effect in TKIR lung cancer. This study provides an insight of GA into understanding anti-tumorigenic function and further overcoming TKI-resistance in advanced lung cancer.

## RESULTS

### Reverse therapeutic effect of gefitinib and GA in NSCLC

**Figure 1:**
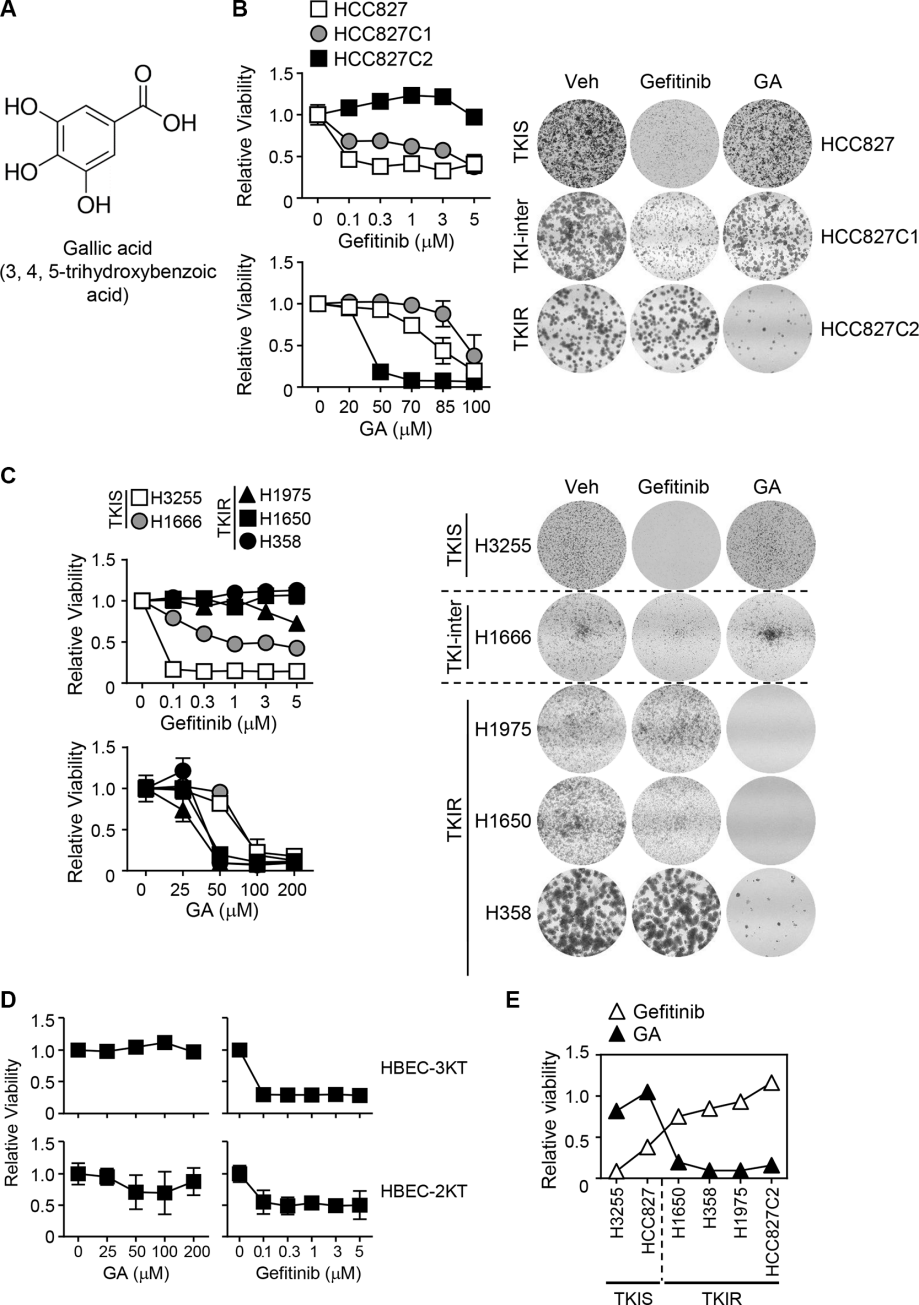
Therapeutic selectivity of GA for gefitinib resistant NSCLC (**A**) A chemical structure of 3,4,5-trihydroxybenzoic acid or GA. (**B**,–**D**) Therapeutic effects of GA and gefitinib were evaluated using MTT as well as colony formation assays in HCC827 clones (B), various NSCLC cells (C) and HBECs (D). Lung cells were treated with gefitinib 0.3 μM or GA 50 μM in colony formation asssay or at indicated concentrations in MTT assay. NSCLC lines were classified into 3 categories upon TKI sensitivity as follows: TKI-sensitive (□), HCC827, H3255; TKI-intermediate (◘), HCC827C1, H1666; and TKI-resistant (■), HCC827C2, H1650, (▲), H1975, (●), H358. In every MTT assay, values are mean ± SEM of five replicate assays. (**E**) The association between GA and gefitinib sensitivity in NSCLC was evaluated using Pearson analysis (*r*^2^ = 0.72, *p* = 0.031). The growth response between GA and gefitinib was negatively correlated to each other in the same panel. The plot represents for growth responses of each cell at gefitinib (0.3 μM) and GA (50 μM) from (B) and (C).

To execute biological studies for TKI-resistance in lung cancer, we have established a panel of HCC827 NSCLC clones upon TKI-sensitivity and further collected five NSCLC cell lines containing different kinds of activating mutations in EGFR as previously described [21–23]. The HCC827 panel consists of parental HCC827 and two independent clones, HCC827C1 and HCC827C2, isolated from the parental one upon TKI-sensitivity for further studies. Using MTT cell growth assay, we first determined drug sensitivity to EGFR inhibitors, gefitinib and erlotinib, in the NSCLC panel and classified into 3 groups upon TKI-sensitivity. Included in each group are TKIS cell lines, HCC827 and H3255; TKI-intermediate responsive lines, HCC827C1 and H1666; TKIR lines, H1650, H358, H1975 and HCC827C2 (Table 1). Note that a chemical structure of 3, 4, 5-trihydroxybenzoic acid or GA was represented (Figure 1A). To explore therapeutic effect of GA in the TKIR lung cancer, we carried out cell growth assay by treating parental and TKIR HCCC827 cells with GA or gefitinib in a dose-dependent manner. Gefitinib showed strong growth inhibition in the parental HCC827 lung cancer but no growth inhibitory effect in the gefitinib-resistant HCC827C2 as expected. However, interestingly, GA strongly inhibited cell growth of the TKIR clone HCC827C2 but not the TKIS parental HCC827 cells (Figure 1B). Consistently, liquid colony formation assay revealed the TKI sensitivity is reversely correlated with GA in the HCC827 cell panel (Figure 1B). In line with HCC827, the intermediately TKI-responsive HCC827C1 line showed less growth inhibitory response to GA treatment compared to HCC827C2 (Figure 1B). Next, to confirm the reverse therapeutic correlation of GA and gefitinib, we further evaluated growth inhibitory effect of both chemicals in seven lung cells including two human bronchial epithelial cell (HBEC) and five NSCLC lines. Note that HBECs are normal cell lines immortalized with CDK4 and hTERt as previously described [24] and the genetic and pharmacological features of the other lung cancer cells were described in detail (Table 1). Indeed, two independent cell viability assays, MTT and colony formation, confirmed that the TKIR cell lines including H1975, H1650, H358 were GA-sensitive but resistant to the gefitinib treatment and vice versa in the TKIS cell lines (Figure 1C). In detail, the cell viability assays revealed that cell proliferation of TKIR H1975, a lung cancer harboring secondary T790M mutation in the kinase ATP binding pocket, were robustly reduced by 50 μM of GA treatment. Similarly, GA successfully inhibited the cell proliferation of H1650 that is insensitive to TKIs due to phosphatase and tensin homologue (PTEN) loss. In addition, H358 cell growth, intrinsically resistant to any TKIs potentially due to K-ras mutation, was dramatically suppressed by GA. However, the TKIS cells, H3255 and H1666, showed no significant growth inhibitory response under the same treatment condition of GA as in TKIR. More importantly, GA showed no toxicity in HBEC2KT and HBEC3KT, which were affected by gefitinib even at 0.1 μM (Figure 1D). The overall summary represents the significant reverse correlation between GA and gefitinib sensitivity in the panel of NSCLC cells (Figure 1E). Taken together, this data highlight the promising effect of GA to overcome TKI-resistance in lung cancer.

**Table 1:** TKI sensitivity of lung cancer cell lines

Cell line	Genetic status	Gefitinib IC_50_ (μM)	Erlotinib IC_50_ (μM)
TKI-sensitive			
HCC827	GEFR^E746_A750del^	0.1	0.1
H3255	EGFR^L858R^	0.1	0.1
TKI-intermediate			
H1666	EGFR^WT^, BRAF^G466V^	1.6	1.1
HCC827C1	EGFR^E746_A750del^	2.5	4.6
TKI-resistant			
H1650	EGFR^E746_A750del^, PTEN^loss^	12.4	16.5
H358	EGFR^WT^, KRAS^G12V^	15.4	5.4
H1975	EGFR^L858R, T790M^	21.2	27
HCC827C2	EGFR^E746_A750del^	27	29.7

NSCLC lines were classified into 3 categories upon TKI sensitivity: TKI-sensitive, HCC827, H3255; TKI-intermediate, HCC827C1, H1666; and TKI-resistant, HCC827C2, H1650, H1975, H358 provided with genetic background. Each IC_50_ was measured at least in triplicate, and average values are shown.

### Gefitinib activates Stat3 signaling in EGFR mutant lung cancer cells

**Figure 2:**
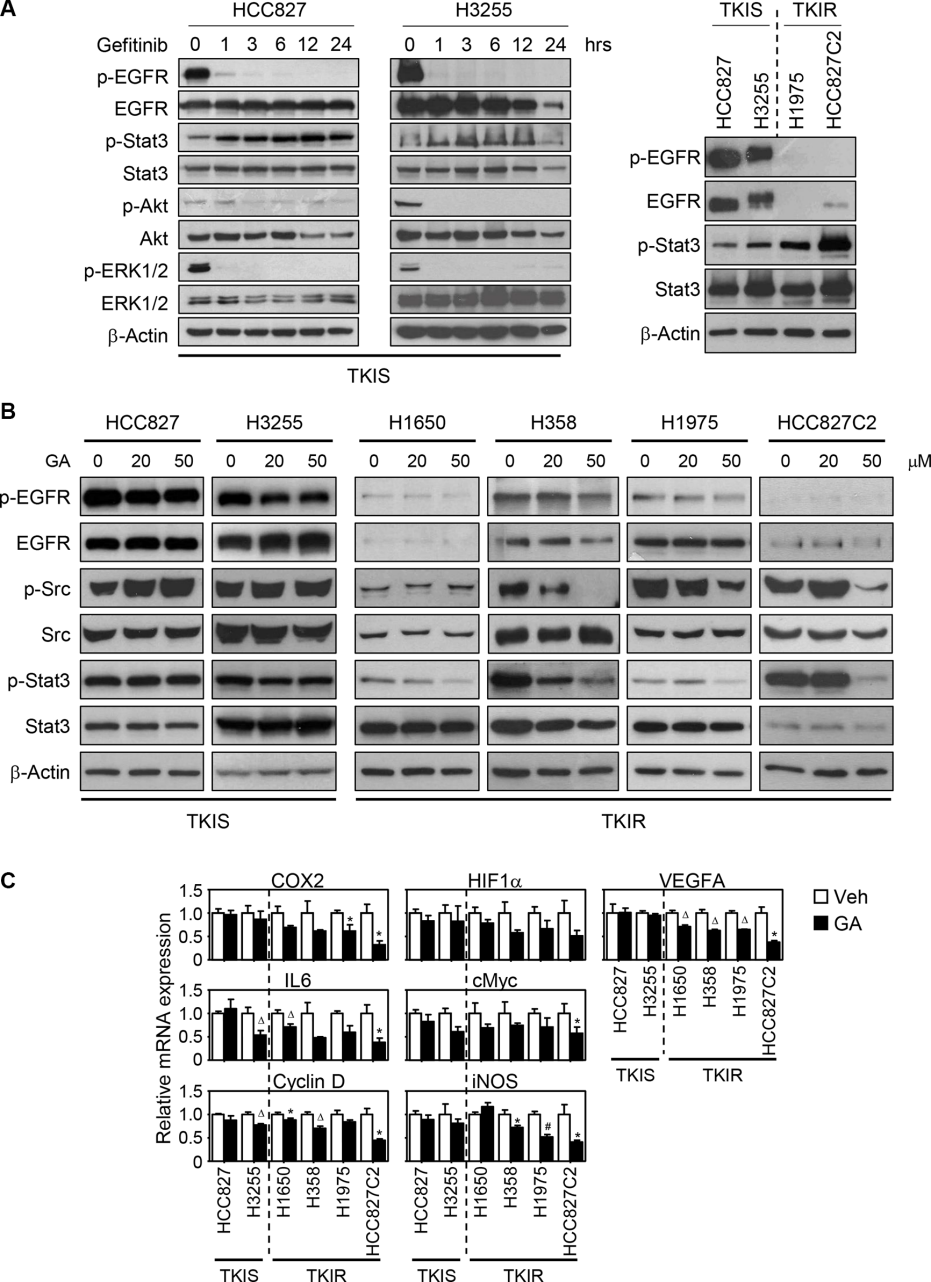
GA inhibits Src-Stat3-mediated signaling in TKIR NSCLC (**A**) Gefitinib treatment induces Stat3 phosphorylation. HCC827 and H3255 cells were treated with 0.3 μM of gefitinib in a time-dependent manner, and followed by immunoblot assay for proteins involved in EGFR and Stat3 signaling. Basal EGFR activation and Stat3 phosphorylation were reversely correlated to each other between TKIS and TKIR cells (right panel). (**B**) GA-mediated Src and Stat3 phosphorylation in TKI-sensitive vs. -resistant NSCLC lines. NSCLC cells were treated with GA (20 μM, 50 μM) for 6 hours and followed by immunoblot assay for phosphorylation of EGFR, Src, and Stat3 proteins. (**C**) mRNA expression of Stat3 regulated genes upon GA treatment. Cells were treated with 50 μM of GA for 24 hours and followed by QPCR assay for mRNA expression of Stat3 target genes. Values are mean ± SEM of triplicate assays. Difference were analysed using Student's *t*-test. * *p* < 0.05; ^Δ^*p* < 0.01; ^#^*p* < 0.001.

Since GA showed TKIR-selective anti-cancer effect, we next wanted to explore the molecular mechanism of GA action in the process. We first investigated the EGFR signal transduction pathways upon gefitinib treatment in both HCC827 and H3255 lung cancer cells. Gefitinib promptly suppressed the EGFR downstream signaling including Akt and ERK1/2 in TKIS cells but showed modest effect in TKIR cells (Figure 2A and Figure S1). Intriguingly, phosphorylation of Stat3 was also induced by gefitinib treatment in as early as 1 hour and maintained over 24 hours after the drug treatment (Figure 2A). Consistently, the Stat3 phosphorylation is basely induced in the TKIR cell lines (H1975, HCC827C2) compared to the TKIS (HCC827, H3255) one (Figure 2A, right panel). Note that TKIS cells have high level of phosphorylated EGFR whereas EGFR activity was down-regulated in TKIR cells possibly due to no longer addiction to EGFR signaling (Figure 2A). This data suggest that Stat3 signaling might play a role in the emergence of acquired resistance during gefitinib exposure. Indeed, a previous report showed that suppression of EGFR signaling induced Stat3 activation in EGFR-mutant but not in EGFR wildtype lung cancer cells. Activated Stat3, subsequently, regulated tumor growth, enabling cancer cells to survive under pressure of targeted therapies [12, 14]. Taken together, Stat3 activation might emerge as an alternative oncogenic bypass and drive cancer cells to escape the EGFR signaling or the TKI suppression. We then determined whether Stattic, a Stat3 inhibitor, could suppress cell growth of TKIR NSCLC cells. To our surprise, Stattic treatment significantly inhibited cell growth of TKIR cells but not of the sensitive ones (Figure S2). Accordingly, targeting Stat3 pathway might be a promising strategy to overcome the TKI resistance in NSCLC.

### GA inhibits Src-Stat3-mediated signaling specifically in TKIR lung cancer cells

As a recent study reported that GA suppressed lipopolysaccharide-induced nuclear factor-kB signaling, resulting in decreased production of IL-6 [20], we wondered if GA inhibits Stat3 phosphorylation and subsequently suppresses Stat3-mediated tumor proliferation, especially in TKIR cells. Thus, we examined the GA effect on Stat3 signaling in two sets of NSCLC lines, TKIS and TKIR cells. To our surprise, GA dramatically suppressed Stat3 phosphorylation at tyrosine 705 in TKIR cells, but not in TKIS cells HCC827 and H3255, in a dose-dependent manner (Figure 2B). The suppression of Stat3 phosphorylation by GA was maximized at 6 hours and maintained up to 24 hours after GA treatment (Figure 2B and Figure S3). Interestingly, EGFR activation, known as one of the upstream pathways for activating Stat3 signaling, was not significantly affected by GA treatment in both TKIS and TKIR cells, suggesting that GA inhibition of Stat3 may not be directly linked to the EGFR regulation (Figure 2B). As an alternative and well-known direct upstream factor for Stat3 activation, we next examined if GA modulates oncogenic Src activity that is involved in tumor progression, metastasis, and angiogenesis [25, 26]. Indeed, GA inhibited Src phosphorylation at tyrosine 416 specifically in TKIR cells but not in TKIS ones (Figure 2B). Furthermore, since Stat3 is a well-known transcription factor that directly regulates a subset of tumor-promoting genes including oncogenic transcription factors, cancer immune-surveillance, angiogenesis, apoptosis, and cell cycle factors [27], we then sought to investigate whether GA regulates Stat3-target genes involving HIF1α, cMyc, COX2, iNOS, IL6, VEGFΑ, and cyclin D. Indeed, GA modestly suppressed the expression of Stat3 target genes, especially in TKIR cells (Figure 2C). This finding highlights that GA mediated Src-Stat3 inhibition and thus resulting in suppression of Src-Stat3-mediated tumor promoting genes to overcome Stat3 addicted TKIR lung cancer.

### GA induces apoptosis and cell cycle arrest specifically in TKIR lung cancer

**Figure 3:**
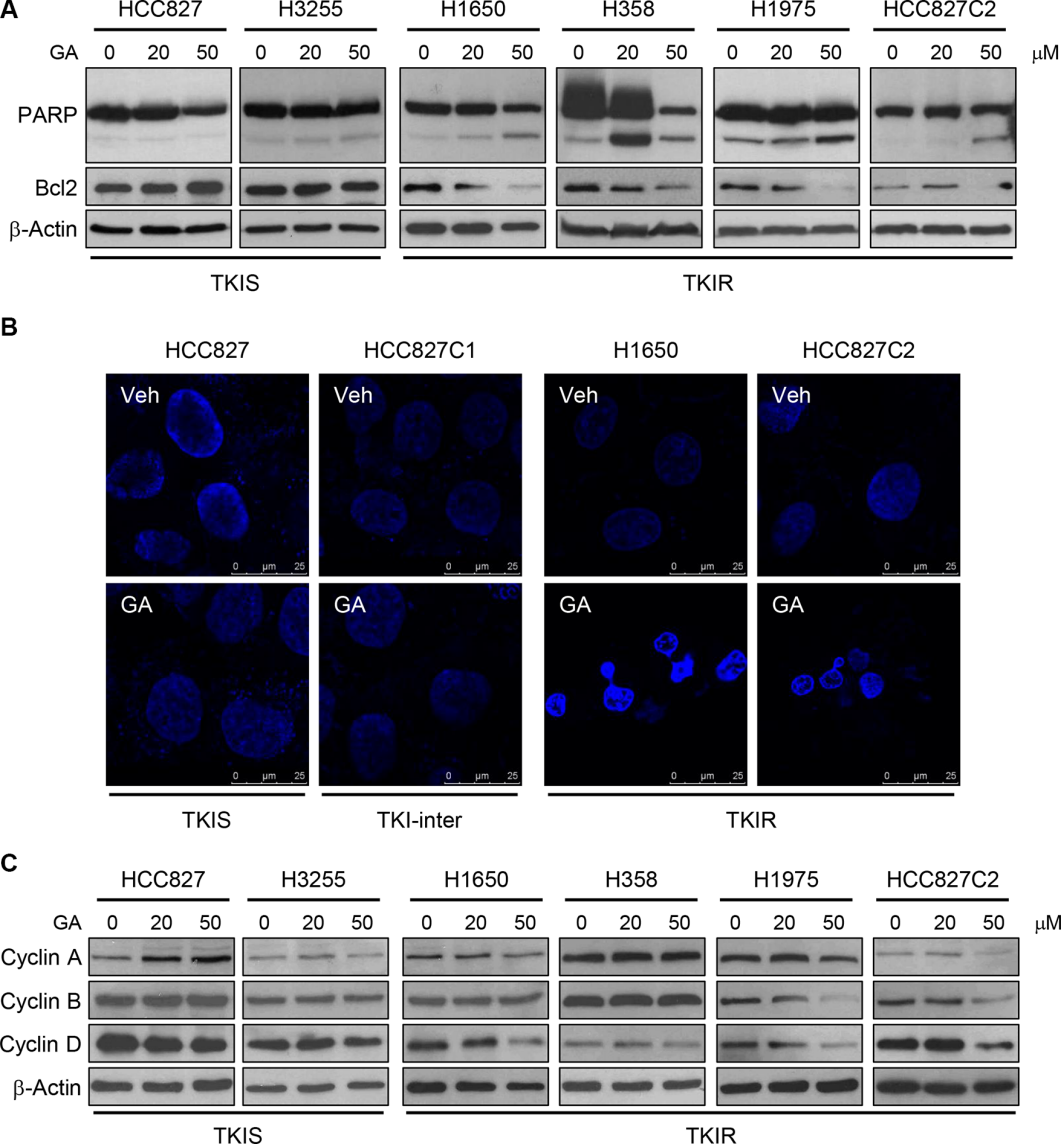
GA induces apoptosis and cell cycle arrest in TKIR NSCLC (**A**, **C**) NSCLC cells were treated with vehicle or GA (20 μM and 50 μM) for 24 hours and immunnoblot assays were performed for the expression of proteins involved in apoptosis (A) and cell cycle regulation (C). (**B**) DAPI staining were performed to determine apoptosis. Cells were treated with 50 μM of GA treatment for 24 hours and followed by DAPI staining.

Since Stat3 activation results in overall cancer cell survival and suppression of apoptosis, we thus examined proteins involved in apoptosis and cell cycle progression in lung cancer treated with GA. As shown in Figure 3A, GA treatment dramatically suppressed anti-apoptotic factor Bcl2 expression while induced cleavage of poly ADP-ribose polymerase (PARP), an apoptotic marker. Consistently, GA treatment induced apoptotic bodies indicated by condensed chromatin and fragmented nucleus in TKIR cells (Figure 3B). In addition to apoptosis induction, GA treatment markedly decreased protein expression of cell cyclins (A, B, D) involved in cell cycle progression in the TKIR lung cancer panel but not in the TKIS one (Figure 3C). Taken together, these results suggest that GA exerted anti-cancer effect specifically in TKIR cells by inhibiting Src-mediated Stat3 phosphorylation, downregulating Stat3 target genes, especially Bcl2 and cyclin D, hence inducing apoptosis and cell cycle arrest.

### GA selectively suppresses TKIR tumor growth *in vivo*

**Figure 4:**
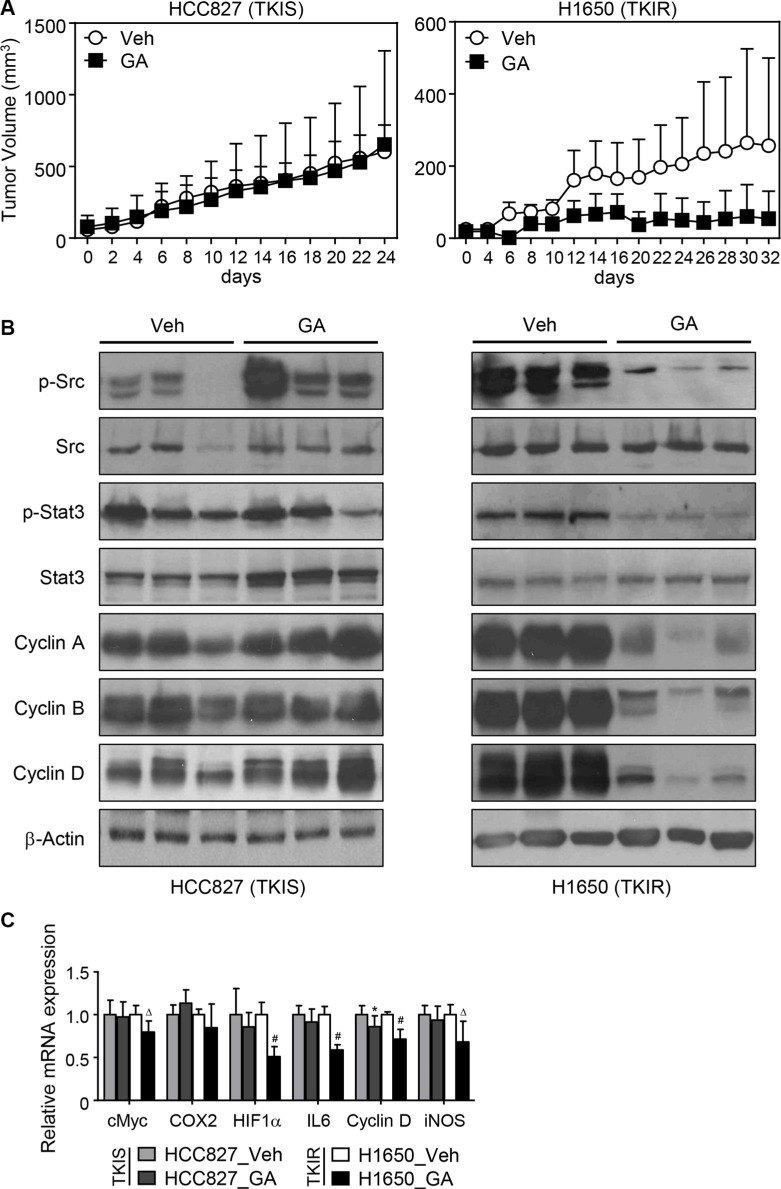
*In vivo* therapeutic evaluation of GA in xenograft model of TKIR tumor *In vivo* xenograft tumor model was established by injecting HCC827 or H1650 lung tumor cells into the flank region of athymic nude mouse. HCC827 and H1650 represent TKI-sensitive and -resistant tumors, respectively. The xenografted mice were intraperitoneally injected with vehicle (*n* = 5, HCC827 and *n* = 4, H1650) or GA (200 mg/kg/day) (*n* = 8, HCC827 and *n* = 6, H1650) every other day for 24 days (HCC827) or 32 days (H1650). Tumor volumes were measured every other day. Tumor volume represents the tumor size ± SEM and statistical analysis was determined using mixed model, *p* = 0.0211 (**A**). At the end of the experiment, tumor tissues were isolated for immunoblot assay (**B**) and QPCR assay (**C**) for genes of interest involving cell cycle regulation and Stat3 signaling. Values are mean ± SEM. Difference were analysed using Student's *t*-test. * *p* < 0.05; ^Δ^*p* < 0.01; ^#^*p* < 0.001.

To confirm the *in vitro* study using *in vivo* model, the xenografted tumor model for HCC827 and H1650 lung cancers was established in the flank region of athymic nude mice and followed by 200 mg/kg of GA or vehicle administration intraperitoneally every other day (Figure 4A). The tumor volume was also measured every other day as described in materials and methods. We found that GA treatment induced dramatic regression of the TKIR xenograft H1650 tumor but not the TKIS xenograft HCC827 tumor (Figure 4A and Figure S4A, S4B). Consistent with the TKIR tumor regression, the expression of cell cyclins were robustly decreased in the GA-treated TKIR xenograft tumor tissues. Furthermore, GA treatment inhibited phosphorylation of Src and Stat3 further leading to the decreased expression of Stat3 target genes including IL6, HIF1α, COX2, cMyc, iNOS, and cyclin D, in the H1650 xenografts but not in the HCC827 xenografts (Figure 4B, 4C and Figure S4C). Taken together, these findings confirm the role of GA, as a potential Src inhibitor, that may overcome TKI resistance in NSCLC *in vivo*.

## DISCUSSION

Targeting oncogenic driver EGFR using reversible or irreversible TKIs has clinically benefited for a subset of lung cancer patients with EGFR mutations [3, 28, 29]. However, TKI-treated lung cancer eventually acquired drug resistance by evolving diverse biological escape mechanisms: secondary mutation T790M or alternative oncogenic bypass tracks to the TKIs. Thus, molecularly targeted therapeutic approach remains a remarkable clinical challenge both effectively and economically, especially in TKIR NSCLC [3, 4, 30]. This requires novel therapeutics or screening new candidate compounds to be developed for overcoming TKIR in lung cancer as well. In this regard, we found GA would become one of the strong candidates to be developed into a lead compound or anti-cancer cocktails with other drugs for overcoming the TKIR lung cancer. GA is a polyphenolic phytochemical commonly found in tea, berries, fruits, and other plants. Notably, growing evidence show that many dietary polyphenols including curcumin, resveratrol, EGCG, and GA exert beneficial chemopreventive effects against various types of cancers *in vitro* and *in vivo* [15, 18, 31, 32].

In the present study, to demonstrate that GA selectively suppresses the TKIR tumor growth and to identify the underlying molecular mechanism of GA action in the process, we have performed several independent cellular and molecular approaches: 1) to investigate GA effects in the TKIR vs. TKIS lung cancer panels including both *in vitro* and *in vivo* systems; 2) to interrogate the molecular pathways for the TKIR-selective anti-tumorigenic function of GA; and 3) to assess GA cytotoxicity in a subset of normal lung cells so that cellular evidence be provided to avoid the potential side effect of GA, if any, for drug development in the future.

To that end, we first utilized an isogenic panel of HCC827 cells consisting of TKI-sensitive, -intermediate, and -resistant clones, which to carefully exclude the potential influence of other genetic backgrounds in the TKIR-selective GA effect. In addition, the TKIR-selective growth inhibitory effect of GA was evidently confirmed in the NSCLC panel consisting of TKIS and TKIR cells, and *in vivo* xenograft model as well. From the comparative analysis for growth inhibitory responses, we notably identified the significant reverse correlation between GA and gefitinib as shown in Figure 1E. However, even with the TKIR-sensitivity to GA, interestingly note that the TKIS cell lines also showed modest growth inhibition only in high doses of GA treatment (Figures 1B, 1C and Figure S6). Thus, it might be intriguing to see if GA would effectively increase therapeutic potential of TKI in EGFR lung cancer. In fact, GA showed additive effect in HCC827C1 cells when treated in combination with gefitinib (Figure S5).

Secondly, from the literature survey for the TKIR mechanisms published and accordingly extensive molecular approaches, we identified that GA inhibition of Src-Stat3 dependent signaling is one of the underlying molecular mechanisms for the TKIR-selective anti-tumorigenic effect. Activation of Stat3 signaling is previously reported as one of the EGFR downstream signaling cascades responsible for the acquired resistance in NSCLC [14, 33]. Indeed, our data also showed that basal level of Stat3 phosphorylation was upregulated in the TKIR lung cancer cells compared to the corresponding TKIS one, suggesting the involvement of Stat3 signaling in the TKI-resistant mechanism. Moreover, although gefitinib treatment successfully inhibited EGFR phosphorylation and followed by reduced activity of downstream signaling such as AKT or ERK1/2, Stat3 phosphorylation was reversely induced upon TKI treatment in TKIS cell lines. This implies that TKI suppression of EGFR signaling might result in a feedback activation of Stat3 signaling and thus its tumor-promoting activity as well. Consistently, treatment of Stattic, a Stat3 inhibitor, dramatically suppressed cell viability in TKIR cells while sparing TKIS cells (Figure S2). On the other hand, Src has been previously reported to induce TKI resistance in EGFR mutant lung cancer by inducing EMT and cancer metastasis [34, 35]. Importantly, phosphorylation of Src and Stat3 have been reported to be upregulated in clinical samples and associated with poor prognosis as well as tumor recurrence [13, 14, 36]. However, Src involvement in Stat3-mediated TKI resistance remains unclear in NSCLC. To that point, we found that both *in vitro* and *in vivo* treatment of GA also suppressed oncogenic Src, an upstream signaling of Stat3, in the TKIR lung cancer but not in the TKIS lung cancer. This is the first report that GA inhibits Src-mediated Stat3 signaling contributing to the TKI resistance in lung cancer. Thus, one might think if GA would be included in TKI-cocktailed therapeutic resume so that the TKI activation of Stat3 signaling would be prevented by GA from the beginning of the treatment. Furthermore, Stat3 plays a critical role not only in the TKI-resistant lung cancer, but also in other multi-drug resistant malignancy including non-Hodgkin's lymphoma, multiple myeloma, epidermoid cell skin carcinoma, prostate carcinoma, and even self-renewal of cancer stem cells [37–41]. This suggests that clinical utility of GA may not be restricted but expanded to various multi-drug resistant cancers.

Lastly, we found the TKI-selective anti-tumorigenic effect of GA accompanied no cytotoxicity in a normal human bronchial epithelial cell line HBEC2KT and HBEC3KT while gefitinib did. Consistently, a previous study has shown GA selectively suppresses oral squamous carcinoma cells without damaging normal human oral keratinocytes [42]. This data is highly encouraging in developing GA into a potential anti-cancer drug, considering that safety and cellular toxicity issues have always become a main pharmacological concern in the drug development process. In this regard, GA could be a good candidate compound to be developed into oral supplements as for cancer chemoprevention.

Collectively, our work supports the idea that combined treatment of GA and gefitinib could provide a promising strategy to prevent acquired resistance upon long-term TKI treatment, and further to develop chemopreventive as well as therapeutic approaches for multi-drug resistant cancer clinics in the future.

## MATERIALS AND METHODS

### Cell culture and reagents

Lung cell lines HBECs, HCC827, H1650, H1975, H358, H1666 and H3255 cells were kindly provided by John D. Minna (University of Texas Southwestern Medical Center, Dallas, Texas, US). All cell lines were re-authenticated by morphology provided by ATCC and by MTT assay to confirm that TKI sensitivity of cells is consistent with previous reports [23, 34]. Lung cancer cell lines were cultured in RPMI 1640 medium supplemented with 5% fetal bovine serum (FBS), 50 U/mL penicillin, and 50 U/mL streptomycin, at 37^°^C, 5% CO_2_ atmosphere. The immortalized HBECs were maintained in K-SFM supplemented with 50 μg/ml of bovine pituitary extract without epidermal growth factor. Cells were periodically tested for mycoplasma contamination. Gefitinib (sc-202166), erlotinib (sc-396113) and stattic (sc-20818) were purchased from Santa Cruz. GA was from Sigma (G7384).

### MTT assay

Cells were splitted into 96-well plates containing cell culture medium in final volume of 100 μL/well and treated as indicated with gefitinib or GA or stattic. The numbers of cells were optimized to avoid over confluence during the assay, as follows: HCC827C2 and H358: 10^3^ cells/well; HCC827, HCC827C1, H1650, and H1975: 2 × 10^3^ cells/well; H1666, H3255, HBEC-2KT and HBEC-3KT: 3 × 10^3^ cells/well. 5 days after treatment, cell viability was assayed using Thiazolyl Blue Tetrazolium Blue (MTT) from Sigma (M2128). All MTT assays were carried out with 5 replications for each data point, and the mean and s.d. calculated.

### Colony formation assay

Cells were splitted into 6-well plates containing cell culture medium in final volume of 2 mL/well and treated with gefitinib or GA or stattic as indicated. Optimized number of cells seeded as follows: HCC827C2 and H358: 3 × 10^3^ cells/well; HCC827, HCC827C1, H1650, and H1975: 5 × 10^3^ cells/well; H1666, H3255, HBEC-2KT and HBEC-3KT: 10^4^ cells/well. 10 days after treatment, colonies were visualized by staining with 0.4% methylene blue solution in 50% methanol.

### Immunoblot analysis

Whole cell lysates and homogenized tumors were prepared in lysis buffer (150 mM NaCl, 1% tritonX-100, 0.5% sodium deoxycholate, 0.1% SDS, 50 mM tris pH 8), containing protease inhibitor (Sigma) and phosphatase inhibitor (Sigma). Protein concentration was determined by BCA protein assay (Pierce) and protein lysates were then reduced and denatured by boiling for 5 minutes at 100^°^C in SDS-sample buffer (50 mM tris pH 6.8, 2% SDS, 0.02% bromophenol blue, 10% glycerol, 1% beta-mercapto ethanol). The lysates were then loaded on SDS-PAGE gels, subsequently transfered to nitrocellulose membrane (BioTrace). Transfer of proteins to membrane was checked by Ponceau staining. The membranes were blocked in 5% skim milk in tris buffer saline containing 0.1% tween 20 (TBST) for 1 hour at room temperature and then incubated with primary antibodies overnight at 4^°^C at manufacturer recommended concentration. The membranes were next washed in TBST and incubated with appropriate secondary antibodies at room temperature following manufacturer's instructions for 1 hour. Finally, the membranes were washed in TBST and band images were acquired using X-ray film (Fuji), X-ray developer and fixer (Vivid). Primary antibodies recognizing protein of interest were purchased from Cell Signaling Technology: phospho-Stat3 (Y705) (#9131), Stat3 (#9139), phospho-Src (Y416) (#6943), Src (#2108), phospho-EGFR (Y1173) (#4407), EGFR (#2232), phospho-Akt (T308) (#9275), AKT (#9272), phospho-ERK1/2 (T202/Y204) (#9101), ERK1/2 (#9102), BCL2 (#2872), PARP (#9542), cyclin B (#4135), cyclin D (#2926). Antibody recognizing β-actin (Ab6276) was purchased from Abcam. Antibody recognizing cyclin A (sc-239) was from Santa Cruz. Secondary antibodies were anti-mouse IgG, HRP conjugated, from Abcam (ab6728) and anti-rabbit IgG, HRP conjugated, from Santa Cruz (sc-2030).

### QPCR analysis

Total RNA was prepared from cells or homogenized tumor tissues using TRIzol reagent (Invitrogen). QPCR RT Master Mix (Toyobo) was then used to reverse-transcribe total RNA to cDNA. The mRNA expression for genes of interest was determined by QPCR in an ABI Prism 7900 HT Sequence Detection System (Applied Biosystems). Three replicates of each PCR reactions were carried out using SYBR green real-time PCR master mixes (Life Technologies). Data analysis was performed using delta-delta Ct method with 18S as the reference gene.

### DAPI staining and confocal microscopy

Cells were seeded on coverslips and treated with GA 50 μM for 24 hours. The cells were then fixed in 4% formaldehyde in culture medium for 15 minutes at room temperature and then washed with PBS. Cells were stained with 4′, 6′-diamidino-2-phenylindole (DAPI, Vector #H1200) to identify apoptotic cells by chromatin condensation and nuclear fragmentation. Fluorescence were imaged using a laser scanning confocal microscope (TCS SPE, Leica Microsystems, Wetzlar, Germany). Every experiment was performed in three different coverslips for each group. On each coverslip, pictures were taken from 10 separated regions. Figure 3B showed representative images for each experiment.

### Xenograft experiment

All animal procedures were reviewed and approved by the Institutional Animal Care and Use Committee (IACUC) of Yonsei University (Wonju Campus), IACUC approval number YWC-140417-1.

For H1650 or HCC827 xenografts, 5 × 10^6^ cells were subcutaneously implanted into the right flanks of 6-week old female Balb/c nude mice. Treatment started when tumors reached ~ 20 mm^3^. Xenografted nude mice were divided randomly into two groups of six mice each (H1650) or eight mice each (HCC827). Mice were treated with either vehicle or 200 mg/kg of GA intraperitoneally every other day for 24 days (HCC827) or 32 days (H1650). Every two days, tumor volumes were determined using digital calipers and calculated using the formula ½ × (width^2^ × length).

### Statistics

Statistical analysis and graphing were performed using Graphpad Prism 6.0 software and Microsoft Excel 2013.

## SUPPLEMENTARY MATERIALS


