# Supplementary material for: Gallic acid inhibition of Src-Stat3 signaling overcomes acquired resistance to EGF receptor tyrosine kinase inhibitors in advanced non-small cell lung cancer
Source: Oncotarget. 2016 Jul 13;7(34):54702–13. doi: 10.18632/oncotarget.10581 (PMC5342374; doi:10.18632/oncotarget.10581)
Supplement: Supplementary file 1 [file oncotarget-07-54702-s001.pdf]

Gallic acid inhibition of Src-Stat3 signaling overcomes acquired resistance to EGF receptor tyrosine kinase inhibitors in advanced non-small cell lung cancer

Supplementary Materials

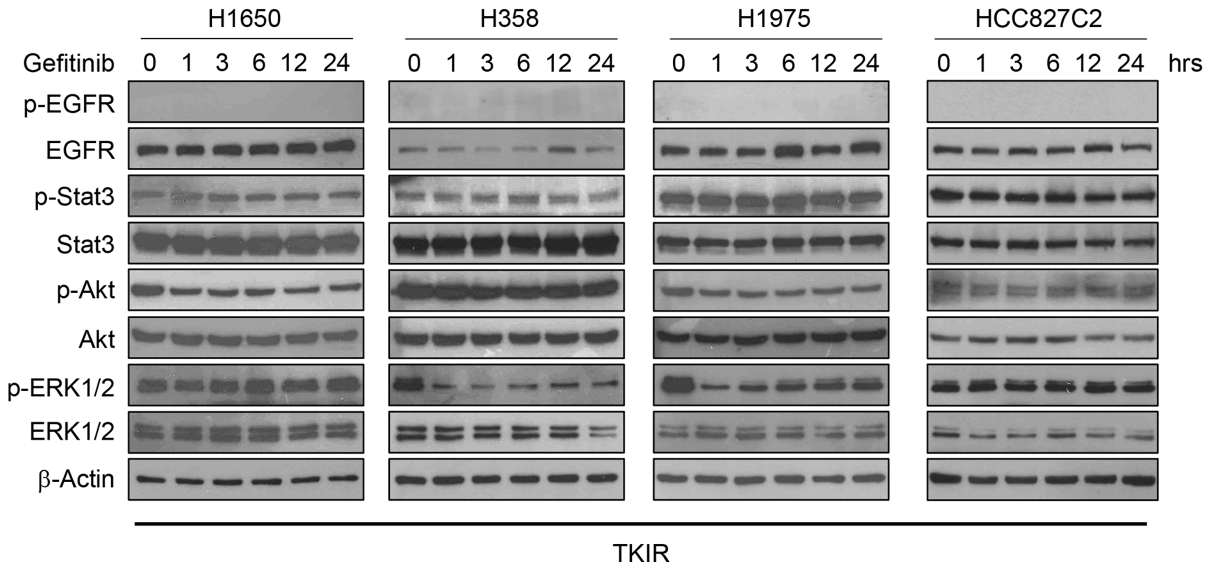

Supplementary Figure S1: Effect of gefitinib treatment on EGFR mediated signaling in TKIR NSCLC cells. TKIR cells H1650, H358, H1975 and HCC827C2 were treated with gefitinib 3 μM in a time-dependent manner and followed by immunoblot assay for phosphorylation of EGFR, Stat3, Akt and ERK1/2.

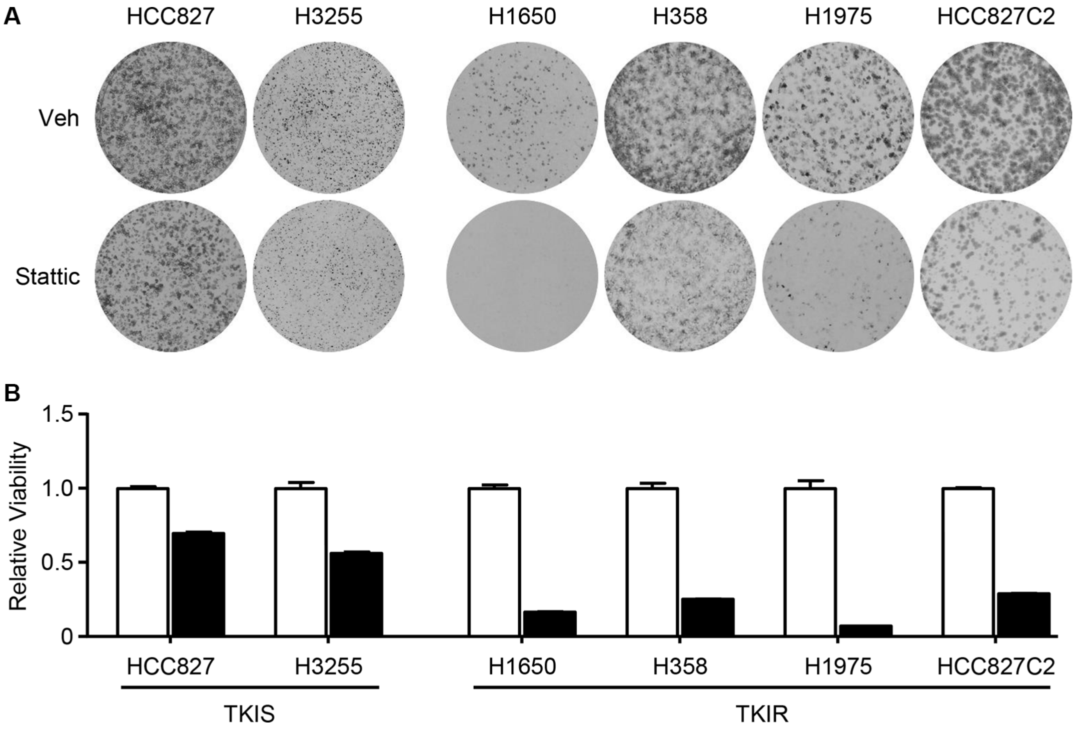

Supplementary Figure S2: Stattic suppresses cell growth of TKIR NSCLC cells. Growth response of NSCLC cells were evaluated using colony formation assay (**A**) and MTT assay (**B**). Lung cancer cells were treated with Stattic 0.3 μM. In every MTT assay, values are mean ± SEM of triplicate assays.

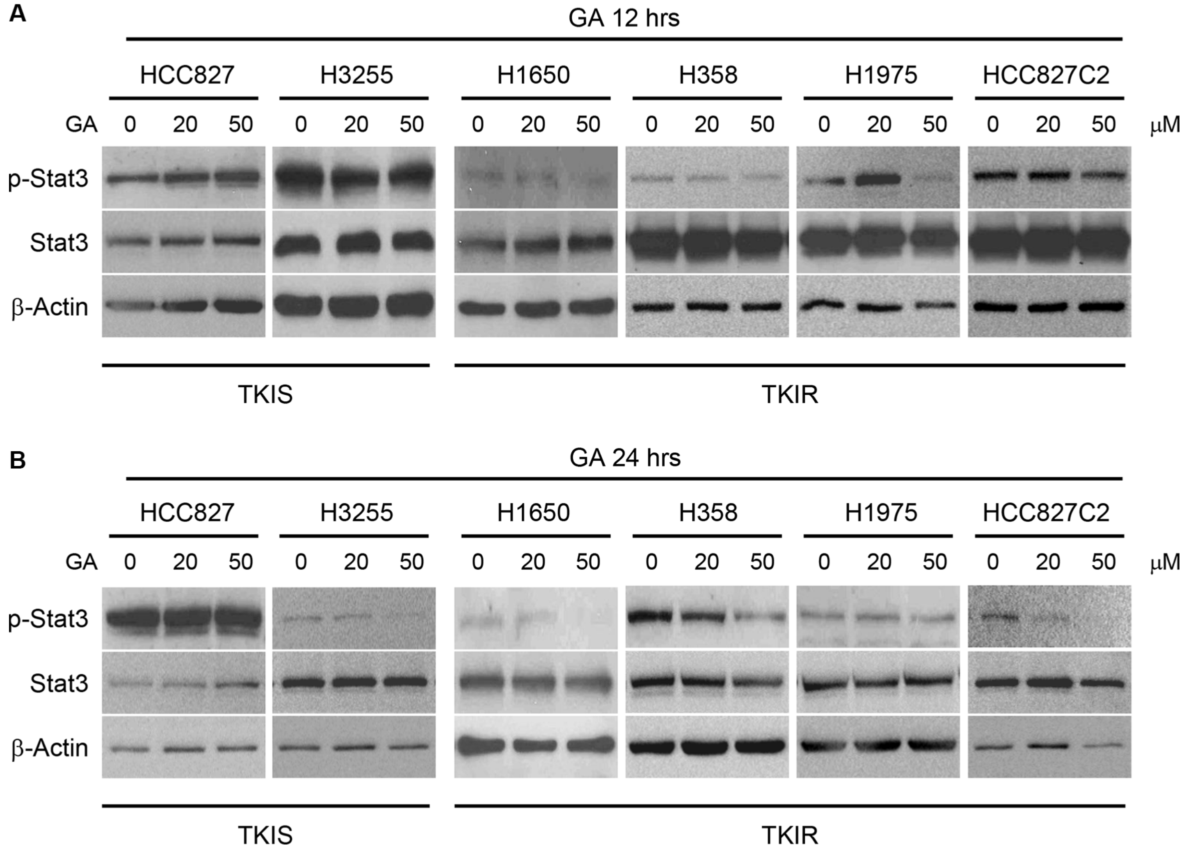

Supplementary Figure S3: GA inhibits Stat3 phosphorylation in TKIR NSCLC cells. (**A**, **B**) GA-mediated Stat3 phosphorylation in TKI-sensitive vs. resistant NSCLC cells. NSCLC cells were treated with GA (20 μM and 50 μM) for 12 hours (A) or 24 hours (B) followed by immunoblot assay for Stat3 signaling.

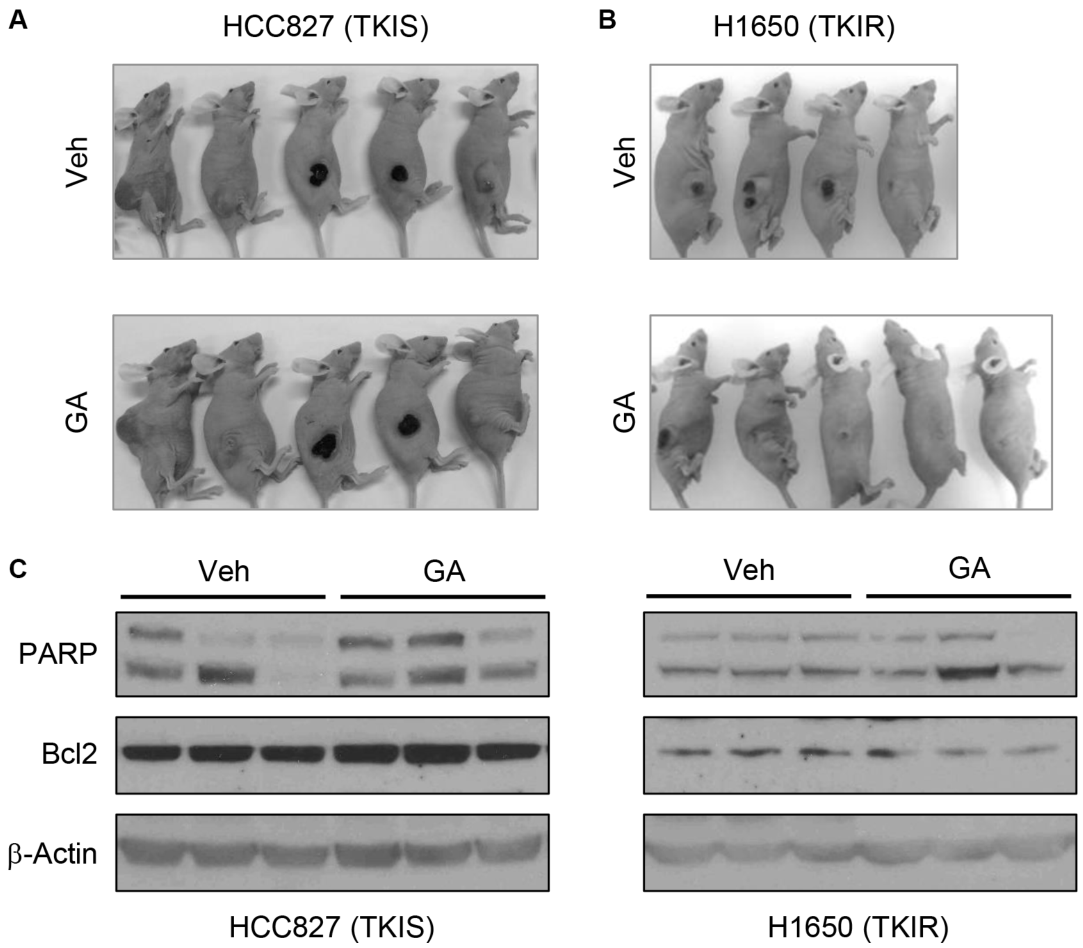

Supplementary Figure S4: GA selectively suppresses tumor growth of TKIR NSCLC *in vivo*. Representative images of tumors from mice treated with vehicle or 200 mg/kg of GA on day 24 (HCC827) (**A**) or day 32 (H1650) (**B**). (**C**) Immunoblot assay was used to evaluate protein expression of apoptotic markers PARP and Bcl2 in tumor tissues isolated from HCC827 or H1650 xenografts treated with vehicle or GA 200 mg/kg.

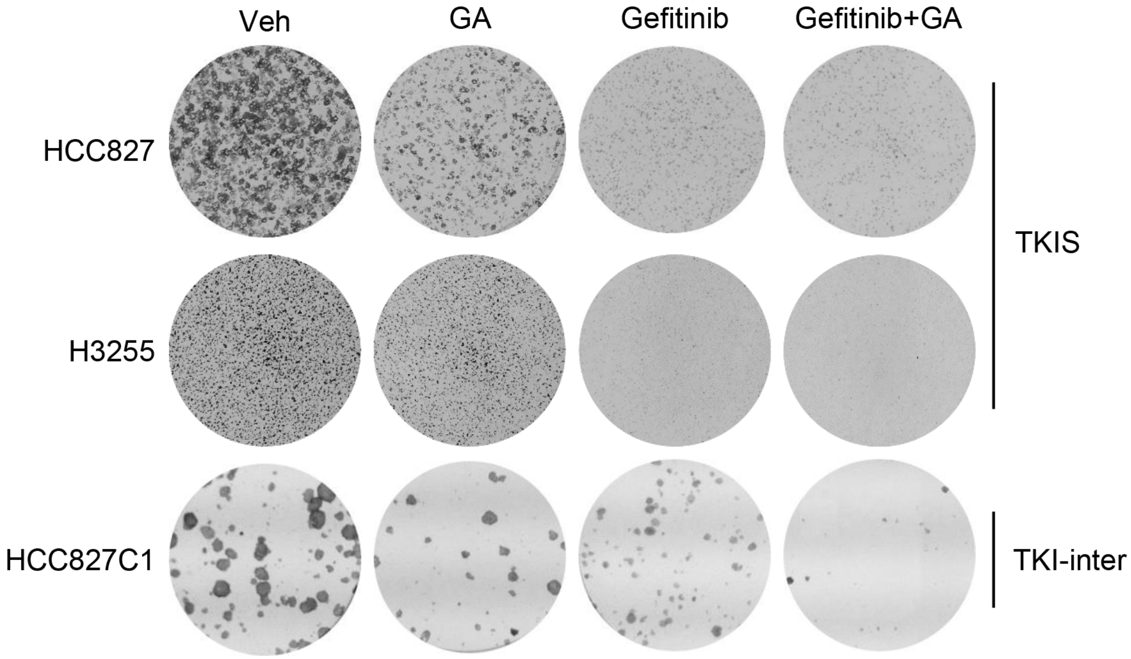

Supplementary Figure S5: Therapeutic effect of combined GA and gefitinib treatment in NSCLC. Combined treatment of GA (50 μM) and gefitinib (0.3 μM) was evaluated using colony formation assays in TKIS cells (HCC827 and H3255) and TKI-intermediate responsive cells (HCC827C1).

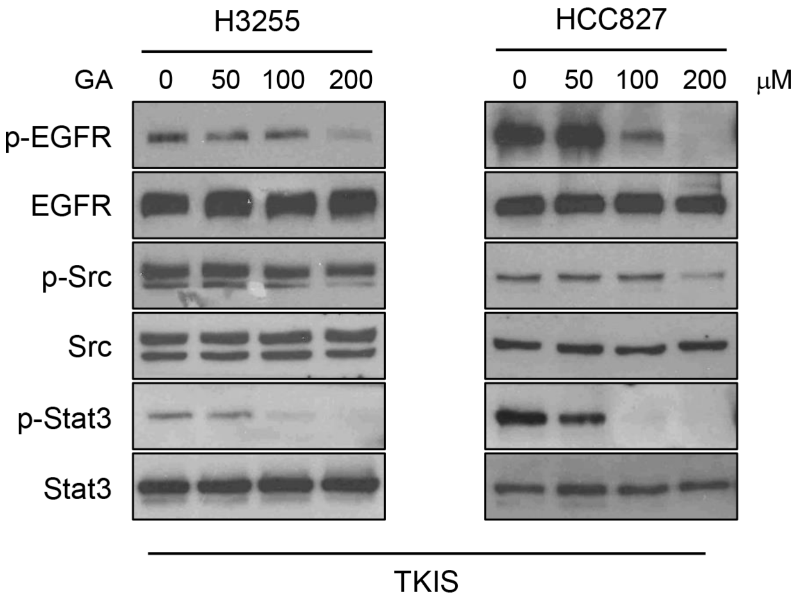

Supplementary Figure S6: High doses of GA suppress Stat3 phosphorylation in TKIS NSCLC cells. TKIS NSCLC cells were treated with GA (50 μM, 100 μM and 200 μM) for 6 hours folowed by western blot for phosphorylation of EGFR, Src and Stat3 protein.

Supplementary Table S1: QPCR primer sequences

Gene	5′-3′ Sequence
COX2-F	AGAAAACTGCTCAACACCGGA
COX2-R	GTGCACTGTGTTTGGAGTGG
Cyclin D-F	CGTGGCCTCTAAGATGAAGGA
Cyclin D-R	CGGTGTAGATGCACAGCTTCT
HIF1α-F	TTTTGGCAGCAACGACACAG
HIF1α-R	TTTTCGTTGGGTGAGGGGAG
IL6-F	CAGTTCCCTGCAGAAAAAGGCAA
IL6-R	ATTTGTGGTTGGGTCAGGGG
iNOS-F	AACCCCAAGGTTGTCTGCAT
iNOS-R	CGTACTGGTCACCACCAACA
cMyc-F	TCTGAGGAGGAACAAGAAGATG
cMyc-R	GTGATCCAGACTCTGACCTTTT
VEGFA-F	CGAGGGCCTGGAGTGTGT
VEGFA-R	ATCCGCATAATCTGCATGGT
